# Un cas d’insuffisance rénale aiguë révélant une endocardite brucellienne et des complications neurologiques à Batna (Algérie)

**DOI:** 10.48327/mtsi.v2i1.2022.229

**Published:** 2022-03-30

**Authors:** Sonia BENAMMAR, Wahiba GUENIFI, Soumia MISSOUM, Chahinez KHERNANE, Fatiha DJEDJIG, Sana BOUKHALFA, Hanane ZOUZOU

**Affiliations:** 1 Service de microbiologie, Centre hospitalo-universitaire Benflis Touhami, Batna, Algérie; 2 Faculté de médecine, Université Batna 2, Batna, Algérie; 3 Service des maladies infectieuses, Centre hospitalo-universitaire de Sétif, Algérie; 4 Faculté de médecine, Université Ferhat Abbas, Sétif, Algérie; 5 Service de néphrologie-hémodialyse et transplantation, Centre hospitalo-universitaire Benflis Touhami, Batna, Algérie; 6 Département de bactériologie, Institut Pasteur d’Algérie, Alger, Algérie; 7 Service de cardiologie, Centre hospitalo-universitaire Benflis Touhami, Batna, Algérie

**Keywords:** *Brucella melitensis*, Endocardite, Complications neurologiques, Insuffisance rénale, Décès, Hôpital, Batna, Algérie, Maghreb, Afrique du Nord, *Brucella melitensis*, Endocarditis, Neurological complications, Renal failure, Death, Hospital, Batna, Algeria, Maghreb, Northern Africa

## Abstract

**Introduction:**

La brucellose est une anthropozoonose endémo-épidémique en Algérie, où elle constitue un problème de santé publique (incidence de 24,41 pour 100 000 habitants en 2017). La maladie est plus invalidante que fatale, les formes graves et les décès sont rares, sauf en cas de localisation cardiaque. Nous rapportons le cas d’un patient atteint d’une brucellose subaiguë multiviscérale compliquée.

**Observation:**

Un homme de 51 ans est hospitalisé en cardiologie pour endocardite, compliquée de manifestations neurovasculaires et cutanées, découverte au stade d’insuffisance rénale sévère, l’une des complications auto-immunes redoutables de l’endocardite infectieuse. Les diagnostics étaient confirmés par différents examens radiographiques et biologiques, l’étiologie brucellienne prouvée par l’examen bactériologique des hémocultures *(Brucella melitensis)* et le sérodiagnostic de Wright.

**Conclusion:**

L’évolution possiblement fatale de cette pathologie doit faire rappeler aux praticiens qu’il faut savoir l’évoquer dans un pays où la brucellose est endémique, agir rapidement et ne pas hésiter à demander au minimum un test sérologique.

## Abstract (en)

**Introduction:**

Brucellosis is a major zoonosis affecting livestock and transmitted to humans; it is widespread worldwide with 500,000 new cases per year according to the World Health Organization. It has become rare in countries that have established an eradication policy of the disease in animals and pasteurization of milk, but remains endemo-epidemic in Algeria, where it constitutes a public health problem (incidence of 24.41 per 100,000 inhabitants in 2017). The disease is more crippling than fatal. Severe forms are exceptional, and deaths are rare, most often following endocarditis or complicated neurological damage. The biological diagnosis is made by culturing the samples (mainly blood cultures), serology or molecular biology methods. We report the case of a patient with complicated and fatal subacute multiorgan brucellosis.

**Observation:**

A 51-year-old man is hospitalized in cardiology for endocarditis, complicated by neurovascular and skin manifestations, discovered in the stage of severe renal failure, one of the dreaded autoimmune complications of infectious endocarditis. The diagnoses were confirmed by various radiographic (echocardiography, brain computed tomography, magnetic resonance imaging) and biological examinations. The brucellian etiology was proved by bacteriological test of blood cultures *(Brucella melitensis)* and Wright’s serodiagnosis, in the absence of the Polymerase Chain Reaction (PCR) test.

**Conclusion:**

Brucellosis can have atypical, multiple, varied and misleading presentations, responsible for a difficult clinical diagnosis. The possibly fatal evolution of this pathology should remind practitioners to evoke it, in particular in front of a multivisceral infectious presentation, in a country where brucellosis is endemic. Clinicians must also act quickly and not hesitate to ask for at least a serological test.

## Introduction

La brucellose est une zoonose majeure frappant le bétail et transmise à l’homme; l’Organisation mondiale de la santé fait état de 500 000 nouveaux cas par an. Elle demeure endémique et pose un problème de santé publique dans le Bassin méditerranéen, le Moyen-Orient, l’Asie, l’Afrique et en Amérique latine [16].

La brucellose humaine est une maladie systémique avec un grand polymorphisme clinique [4]. La maladie est plus invalidante que fatale; les formes graves sont exceptionnelles et les décès sont rares, faisant suite le plus souvent à une endocardite ou une atteinte neurologique compliquée [14].

Nous décrivons une forme polyviscérale et fatale de brucellose humaine, découverte au stade d’insuffisance rénale sévère, complication révélatrice d’une endocardite à *Brucella melitensis* compliquée de localisations méningée et encéphalique.

## Observation

**Figure 1:**
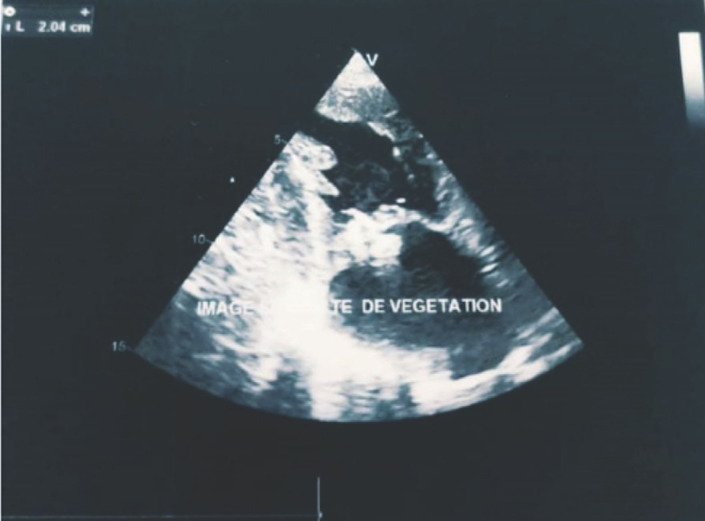
Grosse végétation de 20 mm sur la grande valve mitrale à l’échocardiographie 20 mm thick vegetation on the large mitral valve on echocardiography

**Figure 2:**
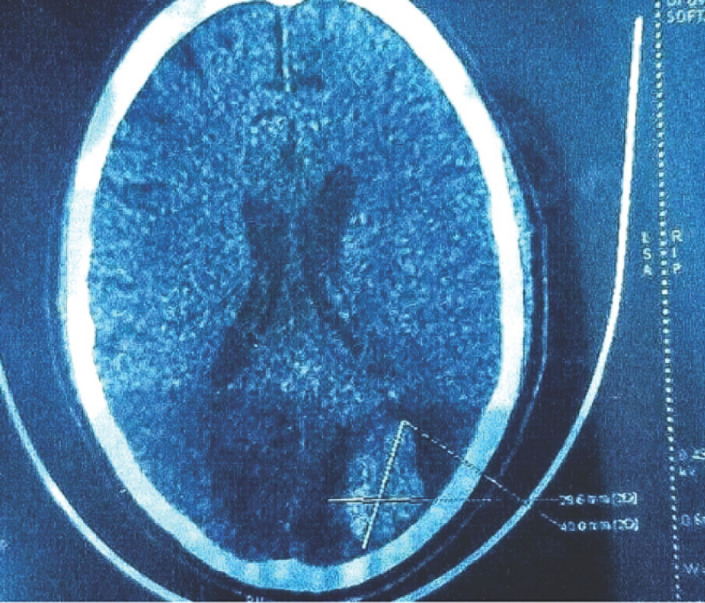
Volumineux hématome occipital gauche (40 mmX29 mm), entouré d’un œdème péri lésionnel à l’examen tomodensitométrique Large left occipital hematoma (40 mm X 29mm) surrounded by peri-lesional oedema on computed tomography examination

**Figure 3:**
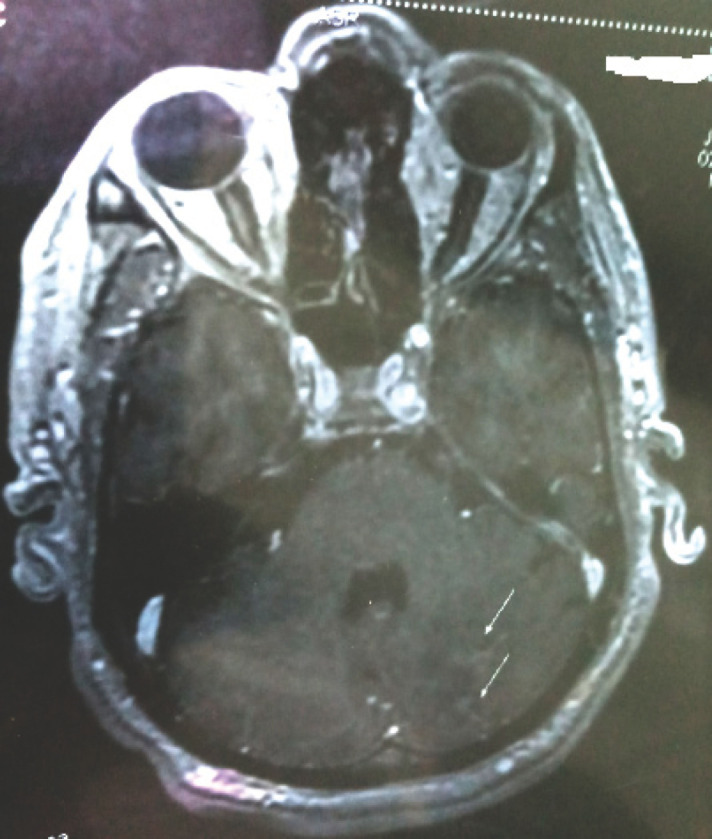
Micro-abcès sous-tentoriels à l’imagerie par résonnance magnétique (IRM) Subtentorial micro-abscesses on magnetic resonance imaging (MRI)

Monsieur K.M. âgé de 51 ans, sans antécédents particuliers, demeurant dans une région rurale, présente depuis 6 mois une altération progressive de l’état général avec fièvre non chiffrée, rachialgies, épistaxis, essoufflement à l’effort ainsi qu’un épisode d’orchi-épididymite unilatérale. Après plusieurs consultations en cabinet de ville sans résultat, un bilan biologique prescrit au 5^e^ mois de l’évolution objectivait une insuffisance rénale aiguë sévère, motif de son orientation en néphrologie où il est hospitalisé pour des séances d’épuration extrarénale. Une glomérulonéphrite rapidement progressive (GNRP) est fortement évoquée (dégradation brutale de sa fonction rénale, chimie des urines et protéinurie des 24 h pathologiques). Devant la présence d’un essoufflement dans un contexte fébrile, une endocardite infectieuse est suspectée, d’où son transfert en cardiologie. À l’admission, il était altéré, fébrile à 38,5 °C; le score de Glasgow était à 10/15, la TA à 110/60 mmHg, la fréquence cardiaque à 75 battements/mn et la saturation en oxygène (SaO_2_) à 95 % en air ambiant. Il présentait une pâleur cutanéo-muqueuse, un purpura pétéchial généralisé, une dyspnée de repos, un souffle d’insuffisance mitrale (IM) de 4/6, une splénomégalie de stade II, une oligo-anurie, une hématurie et une protéinurie (+++). L’échocardiographie transthoracique retrouvait une IM grade III d’apparence ancienne, avec une grosse végétation de 20 mm sur la grande valve mitrale (Fig. 1) et une HTAP (PAPS à 44 mmHg). Le ventricule gauche était dilaté, non hypertrophié, hyperkinétique avec une fraction d’éjection à 71 %. Le bilan biologique retrouvait une CRP à 126,04 mg/l, une procalcitonine à 23,15 ng/ml, une ferritinémie à 550,48 ng/ml, des globules blancs à 10 000 éléments/mm^3^, une hémoglobine à 6,7 g/dl et des plaquettes à 37 000/mm^3^. L’urée sanguine était à 2,4 g/l, la créatinine à 85 mg/l et la protéinurie des 24 heures à 800 mg. Le bilan hépatique retrouvait des transaminases normales, une albumine sérique à 31 g/l et un taux de prothrombine à 57 %. Le diagnostic d’endocardite infectieuse certaine selon les critères de Duke modifiés était retenu, le patient mis sous ceftriaxone et caspofongine (les grosses végétations faisant suspecter une origine fongique), après avoir réalisé trois séries d’hémocultures incubées dans l’automate Bact-Alert^®^. La tomodensitométrie cérébrale objectivait un hématome occipital gauche de 40 mm × 29 mm, entouré d’un œdème périlésionnel ainsi qu’une hémorragie méningée frontale bilatérale et pariétale droite (Fig. 2). La ponction lombaire ramenait un liquide cérébrospinal trouble contenant 584 polynucléaires neutrophiles/mm^3^, une glycorachie à 0,24 (glycémie = 1,57 g/l), mais la culture était stérile (incubation de 72 h seulement). À J4, les hémocultures revenaient positives, permettant l’isolement de *Brucella* spp. L’espèce *Brucella melitensis* était identifiée par technique de lysotypie (lyse par le phage Izanagar et résistance aux phages Tbilissi, Webridge et R/C) au centre de référence à l’Institut Pasteur d’Algérie. La souche était sensible (Tableau I). Le sérodiagnostic de Wright effectué plus tard était positif à 800 UI/ml. L’angiographie-RM cérébrale retrouvait de multiples micro-abcès sus- et sous-tentoriels en rapport avec des localisations d’anévrismes mycotiques multiples et des signes de méningite focale frontale et pariétale droite (Fig. 3 et 4). Le patient était mis sous doxycycline, rifampicine et gentamycine. Il est décédé 2 jours après l’instauration de ce protocole.

**Figure 4:**
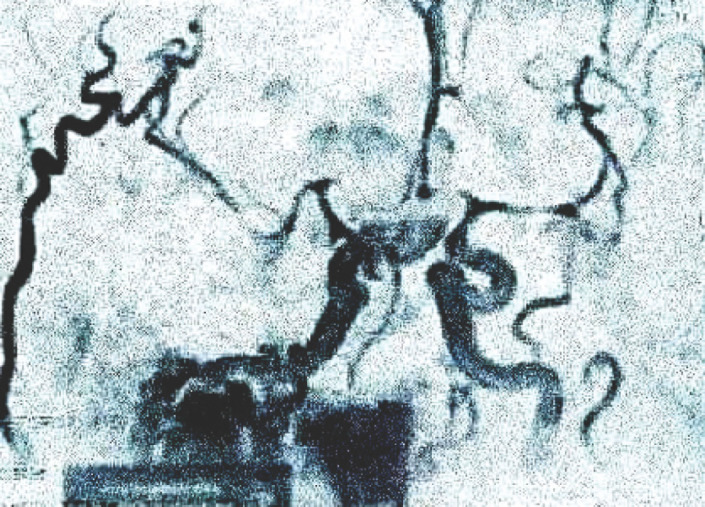
Anévrismes mycotiques à l’angiographie par résonnance magnétique Mycotic aneurysms on magnetic resonance angiography

**Tableau I:** Profil de sensibilité de la souche *Brucella melitensis* aux antibiotiques Antibiotic susceptibility profile of Brucella melitensis strain

Antibiotiques testés	Résultats de CMI (µg/ml)	Interprétation (S, I, R)
Streptomycine	0,5	S
Gentamicine	0,25	S
Doxycycline	0,016	S
Tétracycline	0,023	S
Rifampicine	0,5	S
Triméthoprime/Sulfaméthoxazole	0,003	S

CMI: Concentration minimale inhibitrice. S: sensible, I: intermédiaire, R: résistant

## Discussion

En Algérie, la maladie sévit à l’état endémo-épidémique et constitue un véritable problème de santé publique; l’incidence de la brucellose humaine est passée de 0,36/100 000 en 1989 à 14,15 et 24,41 pour 100 000 habitants en 2014 et 2017 respectivement [9, 16].

À notre connaissance, il s’agit du premier cas publié de brucellose polyviscérale maligne en Algérie.

La brucellose humaine est une maladie systémique avec un grand éventail de tableaux cliniques [4]. Les foyers infectieux peuvent être révélateurs de la maladie, les plus fréquents sont ostéo-articulaires [18] et génito-urinaires à type d’orchi-épididymite souvent unilatérale [4] (une probable localisation chez notre patient). Les atteintes cardiaques ou neurologiques sont moins fréquentes mais graves [3, 13].

Le caractère non spécifique de la maladie fait que la brucellose n’est pas systématiquement évoquée, essentiellement en pratique de ville, d’où l’errance médicale.

Le patient présentait une insuffisance mitrale méconnue, probablement rhumatismale faisant le lit aux greffes bactériennes. La présence des végétations de grande taille s’accompagne d’un risque embolique accru [1], comme ce fut le cas pour notre patient. La taille de la végétation a fait suspecter un agent fongique, conformément à la littérature [8]. Il est aussi décrit que des végétations exubérantes sont corrélées à l’étiologie brucellienne; il s’agit d’une localisation rare touchant seulement 0 à 2 % des patients infectés et ne représentant que 0,5 à 1 % des endocardites infectieuses [2, 12].

Au cours des endocardites infectieuses, les manifestations extracardiaques peuvent toucher de multiples organes et en être révélatrices [15]. Notre patient a été initialement hospitalisé en néphrologie pour insuffisance rénale aiguë dans le cadre d’une glomérulonéphrite rapidement progressive post-infectieuse; les signes cliniques non spécifiques à l’endocardite retardent fréquemment le diagnostic de l’atteinte cardiaque [1, 5]. La brucellose implique rarement le rein [13], cependant l’atteinte de cet organe représente une complication fréquente de l’endocardite [10]. La glomérulonéphrite rapidement progressive est responsable de moins de 1 % des cas d’insuffisance rénale aiguë, et est découverte tardivement [7].

L’atteinte neurologique représente 1 à 7 % des brucelloses et se caractérise par une grande diversité des modes de présentations cliniques [4, 6]. La neurobrucellose représente la première complication des endocardites brucelliennes, elle est d’origine embolique très fréquemment, hémorragique et/ou infectieuse plus rarement [5, 12, 15].

Les anévrismes mycotiques étaient multiples, de grande taille; leur rupture était à l’origine du saignement intracérébral et méningé, ayant contribué à aggraver le pronostic [11, 15, 17].

Le décès du patient est expliqué par la gravité et la multiplicité des atteintes, mais surtout le retard du diagnostic et de l’instauration du traitement. En effet, l’endocardite représente la première cause de décès par brucellose en zone d’endémie [1, 12].

## Conclusion

La brucellose pose encore un problème important économique et de santé publique, surtout dans les régions rurales de l’Algérie, malgré les efforts de lutte déployés contre cette zoonose. Elle peut avoir des présentations très variées et trompeuses, d’où un diagnostic clinique difficile. La brucellose doit être toujours gardée à l’esprit par nos praticiens et recherchée dans le bilan d’une atteinte cardiaque, neurovasculaire, ou en présence d’une atteinte multiviscérale. Des examens sérologiques permettant un résultat rapide et fiable devraient être systématiquement demandés dans ces cas, pour un traitement antibiotique adapté d’emblée.

## Principes éthiques

Le comité consultatif d’éthique du Centre hospitalo-universitaire (CHU) de Batna a donné son approbation pour le projet de publication de cet article.

Nous confirmons avoir travaillé en accord avec les principes énoncés dans les lois internationales qui assurent la protection de la vie privée et les droits des patients.

Les parents du patient ont consenti à l’utilisation des données cliniques et paracliniques de leur proche dans une publication scientifique.

## Remerciements

Les auteurs remercient chaleureusement le Pr H. Tali-Maamar et le Pr S. Hasnaoui de l’Institut Pasteur d’Alger pour leur collaboration (complément du diagnostic microbiologique).

## Liens d’intérêts

Les auteurs ne déclarent aucun lien d’intérêt. La réalisation de cet article n’a fait l’objet d’aucun financement spécifique.

## Contribution des auteurs

S. Benammar a recueilli les données du cas du dossier médical, rédigé l’article et approuvé la version finale à publier. W. Guenifi a examiné et corrigé l’article, puis approuvé la version finale à publier.

H. Zouzou a assuré la prise en charge du patient et approuvé la version finale à publier.

S. Missoum a assuré la prise en charge du patient et approuvé la version finale à publier.

S. Boukhalfa a assuré le diagnostic bactériologique et approuvé la version finale à publier.

F. Djedjig a assuré le diagnostic bactériologique et approuvé la version finale à publier.

C. Khernane a recueilli les données du cas du dossier médical, assuré le diagnostic bactériologique et approuvé la version finale à publier.

Tous les auteurs ont lu et approuvé le manuscrit final.
